# Genetics of fasting and postprandial metabolite levels are overlapping

**DOI:** 10.1152/physiolgenomics.00101.2017

**Published:** 2018-01-26

**Authors:** Ruifang Li-Gao, Renée de Mutsert, Frits R. Rosendaal, Ko Willems van Dijk, Dennis O. Mook-Kanamori

**Affiliations:** ^1^Department of Clinical Epidemiology; Leiden University Medical Center, Leiden, the Netherlands; ^2^Department of Human Genetics, Leiden University Medical Center, Leiden, the Netherlands; ^3^Department of Medicine, Division of Endocrinology, Leiden University Medical Center, Leiden, the Netherlands; ^4^Department of Public Health and Primary Care, Leiden University Medical Center, Leiden, the Netherlands

**Keywords:** metabolites, postprandial, replication, SNPs

## Abstract

In 2015, a genome-wide association study described 59 independent signals that showed strong associations with 85 fasting metabolite concentrations as measured by the Biocrates AbsoluteIDQ p150 kit. However, the human body resides in a nonfasting state for the greater part of the day, and the genetic basis of postprandial metabolite concentrations remains largely unknown. We systematically examined these previously identified genetic associations in postprandial metabolite concentrations after a mixed meal. Of these 85 metabolites, 23 were identified with significant changes after the meal, for which 38 gene-metabolite associations were analyzed. Of these 38 associations, 31 gene-metabolite associations were replicated with postprandial metabolite concentrations. These data indicate that the genetics of fasting and postprandial metabolite levels are significantly overlapping.

## BACKGROUND/MOTIVATION FOR THE STUDY

In 2015, a genome-wide association study described 59 independent signals that showed strong associations with a wide range of metabolite concentrations as measured by the Biocrates AbsoluteIDQ p150 kit (1). However, this study was limited to metabolite concentrations in the fasting state, and the human body resides in a nonfasting state for the greater part of the day. Therefore, our objective was to examine these associations within postprandial metabolite concentrations.

## PHENOTYPE

Fasting blood samples were drawn in 478 middle-aged men and women. Within the next 5 min after the fasting blood draw, a liquid mixed meal [400 ml, 600 kcal, with 16% of energy (En%) derived from protein, 50 En% carbohydrates, and 34 En% fat] was consumed, and subsequent blood samples were drawn 150 min after the meal. Metabolomic measurements were performed in the postprandial EDTA-plasma samples with the Biocrates AbsoluteIDQ p150 assay. The metabolite concentrations were logarithm-transformed to obtain normal distributions.

### 

#### Cohort details.

This study was embedded in the Netherlands Epidemiology of Obesity (NEO) study, a population-based prospective cohort with 6,671 participants from the greater area of Leiden (in the west of the Netherlands) (2). In 478 participants [56% men, mean (SD) body mass index (BMI) 30.4 (5.0) kg/m^2^] the metabolite concentrations were measured 150 min after a mixed meal.

#### Type of study.

Candidate single nucleotide polymorphisms (SNPs).

#### Details of the SNPs studied.

We set out to examine 123 genetic associations (59 independent signals with 85 unique metabolites) with fasting metabolites reported in Draisma et.al (1). on postprandial metabolite concentrations. After we dropped the variants with low imputation quality (imputation info <0.4) and/or minor allele frequency <0.01, 75 gene-metabolite associations remained between 54 postprandial metabolites and 47 unique SNPs for analysis (Supplemental Table S1). (The online version of this article contains supplemental material.)

#### Analysis model.

We used paired *t*-tests to identify the metabolites with significant changes (*P* < 0.05) after the meal. Additive genetic models were used to assess the associations to the postprandial metabolite concentrations by linear regressions, adjusted for age, sex, BMI, and the first four principal components. A *P* value<1.3E-3 (0.05/38, by Bonferroni correction of multiple testing) was considered as a successful replication.

## RESULTS

Of the 54 postprandial metabolites, 23 changed significantly in concentration after the meal, involving 38 gene-metabolite associations. Half of these 38 associations (*n* = 19) previously identified based on fasting metabolites also revealed pronounced associations to postprandial metabolites. For some gene-metabolite associations the effect size was much larger for the postprandial concentration than for the fasting concentration, e.g., rs1171614 with acetyl-L-carnitine (C2) (β_postprandial_ = −0.14, *P* value = 8.45E-12 vs. β_fasting_ = −0.07) and rs2238732 with proline (β_postprandial_ = 0.24, *P* value = 3.64E-11 vs. β_fasting_ = 0.18) (Supplemental Table S2).

## INTERPRETATION

In this study, we replicated half of the previously described gene fasting-metabolite associations in postprandial metabolites after a mixed meal with very high levels of significance. Interestingly, some of the genetic signals were stronger as compared with fasting metabolite concentrations.

## GRANTS

Metabolite measurements were performed with support of the Dutch Science Foundation ZonMW - VENI Grant 916.14.023.

## DISCLOSURES

No conflicts of interest, financial or otherwise, are declared by the authors.

## AUTHOR CONTRIBUTIONS

R.L.-G. and D.O.M.K. analyzed data; R.L.-G. and D.O.M.K. interpreted results of experiments; R.L.-G. prepared figures; R.L.-G. drafted manuscript; R.L.-G., R.d.M., K.W.v.D., and D.O.M.K. edited and revised manuscript; R.L.-G., R.d.M., F.R.R., K.W.v.D., and D.O.M.K. approved final version of manuscript; R.d.M., F.R.R., and K.W.v.D. conceived and designed research.
